# Precise hourly personalized embryo transfer significantly improves clinical outcomes in patients with repeated implantation failure

**DOI:** 10.3389/fendo.2024.1408398

**Published:** 2024-07-15

**Authors:** Yameng Xu, Jing Du, Yangyun Zou, Xiaoli Lin, Yulin Chen, Lan Ma, Shan Jiang, Xiufeng Lin

**Affiliations:** ^1^ Reproductive Medicine Centre, Boai Hospital of Zhongshan Affiliated with Southern Medical University, Zhongshan, China; ^2^ The Second School of Clinical Medicine, Southern Medical University, Guangzhou, Guangdong, China; ^3^ Department of Clinical Research, Yikon Genomics Company, Ltd., Suzhou, Jiangsu, China

**Keywords:** endometrial receptivity, frozen embryo transfer, personalized embryo transfer, recurrent implantation failure, RNA-Seq-based endometrial receptivity test

## Abstract

**Purpose:**

This study investigated whether RNA-Seq-based endometrial receptivity test (rsERT)—which provides precision for the optimal hour of the window of implantation (WOI)—can improve clinical outcomes of frozen embryo transfer (FET) cycles in patients with a history of repeated implantation failure (RIF).

**Methods:**

Patients with a history of RIF who received at least one autologous high-quality blastocyst during the subsequent FET cycle were retrospectively enrolled and divided into two groups: rsERT and FET, comprising patients who underwent rsERT-guided pET (n=115) and standard FET without rsERT (n=272), respectively.

**Results:**

In the rsERT group, 39.1% (45/115) of patients were receptive. rsERT patients showed a higher probability of achieving both positive human chorionic gonadotropin (63.5% vs. 51.5%, P=0.03) and clinical pregnancy (54.8% vs. 38.6%, P=0.003) rates. In subgroup analysis, rsERT patients with non-receptive results had higher clinical pregnancy rates than patients undergoing FET (58.6% vs. 38.6%, P=0.003). rsERT patients with receptive results guided by rsERT with a precise WOI time had higher, although non-significant, clinical pregnancy rates (48.9% vs. 38.6%, P=0.192) than patients who underwent standard-time FET.

**Conclusion:**

Hourly precise rsERT can significantly improve the probability of achieving clinical pregnancy in patients with RIF, especially in those with non-receptive rsERT results.

## 1 Introduction

Successful embryo transfer depends on the molecular synchronization between a well-developed blastocyst and endometrium receptivity (1). Although preimplantation genetic testing for aneuploidy (PGT-A) can be performed to select euploid blastocysts for transfer (2), endometrial factors may still contribute to implantation failure. For instance, uterine and endometrial abnormalities, such as endometrial polyps, endometritis, intrauterine adhesions, thin endometrium, hysteromyomas, and uterine malformations, have been found to adversely affect embryo transfer outcomes (3). Furthermore, many patients still fail to achieve pregnancy despite treatment for these problems. Thus, recent attention has been focused on determining whether endometrial receptivity can improve the reproductive outcomes of embryo transfer.

Endometrial receptivity refers to the specific status of the endometrium to undergo trophoblast invasion. The period of receptivity, termed the “window of implantation” (WOI) (4), generally occurs in the mid-secretory phase. Since the WOI is believed to last for only 2 days (5), transferring embryos at the appropriate time is crucial for successful assisted reproductive technology (ART) treatment. Generally, blastocysts are transferred on day 7, following the luteinizing hormone (LH) surge (LH+7) in the natural cycle; or on day 5, following progesterone supplementation (P+5) in the hormone replacement therapy (HRT) cycle, to synchronize embryo transfer with the WOI. However, this timing is not uniform in all women (6), and some patients may suffer from WOI displacement or disruption; this may result in embryo transfer not being performed at a time when the endometrium is receptive, potentially resulting in implantation failure (7). Repeated implantation failure (RIF) has been reported to affect approximately 10% of patients undergoing ART treatment (8). Although the causes of RIF have not yet been fully elucidated, displacement and disruption of the WOI are thought to be the main aetiologies (9). Therefore, several methods have been used to identify the WOI, including ultrasound and histological examination; however, both methods lack accuracy and objectivity (10).

The endometrial receptivity array (ERA), which has recently been used in clinical practice, can detect whether an endometrial biopsy sample is receptive based on the expression of 238 genes analysed using an artificial intelligence (AI) algorithm (11). Thus, ERA-guided personalized embryo transfer (pET) has been developed, and it aims to synchronize embryo transfer with the WOI to improve the ART treatment outcomes of patients with RIF (12). Although the ERA can classify endometrial samples in consideration of seven possible profiles with a 12-h accuracy (13), its clinical efficacy remains controversial. Several prior studies have shown that the ERA can significantly improve clinical outcomes (14, 15), while others have failed to identify significant differences between standard embryo transfer and ERA-guided pET (16–18). Considering its high cost and uncertain efficacy, an increasing number of questions have been raised regarding its usage (19, 20). Moreover, given that many patients with RIF may suffer from WOI displacement or narrow WOI, more reliable methods are needed to predict the WOI hour-level accurately.

To predict the WOI with higher accuracy and improve clinical outcomes in patients with RIF, an RNA-Seq-based endometrial receptivity test (rsERT) was developed in 2021 (21) and subsequently optimized. Using RNA-Seq technology in combination with an AI learning algorithm, the rsERT can predict the optimal implantation point with hourly precision, rather than a 12-hour window. Therefore, this study was designed to determine whether the application of rsERT to predict the optimal WOI with high accuracy can result in improvements in the treatment of patients with RIF.

## 2 Materials and methods

### 2.1 Study design and participants

This retrospective study was performed on patients with RIF treated at the Reproductive Medicine Centre, Boai Hospital of Zhongshan between January 2020 and December 2022. In this study, RIF was defined as the failure to achieve clinical pregnancy after at least two fresh or frozen embryo transfer (FET) cycles, with at least two morphologically high-quality blastocysts or four high-quality cleavage-stage embryos. Only the first HRT-FET cycle after unsuccessful embryo implantation was included; then, each patient received at least one high-quality autologous blastocyst, according to the Gardner alpha-numeric criteria (defined as more than three expansion stages with at least one ampere) (22) in the subsequent FET cycle.

All enrolled patients were aged <50 years, with normal karyotypes and adequate endometrial thickness (≥7 mm). All patients had undergone hysteroscopy, and patients with any untreated uterine pathologies, such as endometrial polyps, endometritis, intrauterine adhesions, submucous hysteromyomas, intramural hysteromyomas compressing the endometrium, and other factors that could affect the endometrial environment, natural cycles, minimal-stimulation FET cycles, and PGT cycles were excluded. Furthermore, patients who received vaginal progesterone supplementation for endometrial preparation and luteal phase support were excluded. All patients who met the inclusion and exclusion criteria were divided into two groups: Group rsERT, including patients who underwent pET guided by rsERT (n=115), and Group FET, including patients who underwent standard FET directly (n=272). No patients received donated oocytes or embryos in this study.

Institutional review board approval was obtained for this study from the institutional review board of the Boai Hospital of Zhongshan (Application ID: KY-2022-010-14; date of approval: October 2022). All experiments were conducted in accordance with the Declaration of Helsinki. Informed consent was waived owing to the retrospective analysis of anonymized data.

### 2.2 Endometrial preparation and embryo transfer

All patients underwent HRT or gonadotropin-releasing hormone agonist (GnRH-a) HRT cycles to prepare their endometria for rsERT/FET. During these cycles, oestrogen therapy began on menstrual days 2–4 following administration of oral (4–8 mg/day, Progynova; Bayer) or transdermal (5–15 g/day, Jianmin) oestradiol, and the oestrogen dosage was adjusted by the clinician based on the endometrial thickness, which was monitored using two-dimensional vaginal ultrasound every 3-4 days. After 10–14 days of oestrogen administration, the endometrial thickness was measured using a two-dimensional vaginal ultrasound. Additionally, blood oestradiol (E2) and P4 levels were measured to confirm the absence of spontaneous ovulation. If the thickness of the endometrium was ≥7 mm in patients who underwent rsERT, an endometrial biopsy was scheduled; progesterone supplementation was initiated 5 days prior to the procedure with an intramuscular injection of progesterone in oil (40–60 mg/day). During the GnRH-a HRT cycle, triptorelin acetate (3.75 mg, Diphereline; Ipsen) was administered on menstrual days 2–4; after 28 days, HRT treatment commenced, as previously described.

Group rsERT received endometrial preparation treatment as their mock cycle in rsERT, and pET was performed in the next cycle at the best optimal time for implantation, as predicted by rsERT. Meanwhile, group FET received regular HRT or GnRH-a HRT cycles for endometrial preparation, and blastocysts were transferred 5 days following progesterone administration (P+5). All embryo transfers were conducted by experienced physicians under transabdominal ultrasonography guidance.

Pregnancy examinations were carried out 12 days following embryo transfer, and women who became pregnant continued to receive luteal phase support for 2–3 weeks. Then, transvaginal sonography was performed 2–3 weeks following the first pregnancy test to confirm the presence of an intrauterine gestational sac and pregnancy viability. All women who achieved clinical pregnancy continued the progesterone and oestradiol treatments until week 10 of pregnancy.

### 2.3 Receptivity measurement

Differentially expressed genes (DEGs) in different endometrial receptive phases were detected, as previously described (21). Regarding the optimal time prediction of the WOI, three-point samplings from a previous study were applied as a training dataset for model construction; specifically, using the sampling time and corresponding clinical pregnancy outcomes, numerical values with the hourly precision of the expected optimal WOI were defined in these training samples. For example, if three samples from P+3, P+5, and P+7 in one patient were predicted to have pre-receptivity, post-receptivity, and post-receptivity by the previous rsERT method (21), blastocysts were transferred on P+4 (or a day 3 cleavage embryo on P+2). If the patient had an intrauterine pregnancy, the numerical hourly values for these three samples were approximately 24 h, -24 h, and -72 h, respectively. Different combinations generated quantitative labels at 0 h, 24 h, -24 h, 48 h, -48 h, 72 h, -72 h, 96 h, and -96 h for each sample in the training dataset. Furthermore, a random-forest regression model from the Ranger R package (version 0.12.1) was applied to predict the optimal implantation point with hourly precision (23). The infinitesimal jackknife resampling method was then applied to estimate the standard errors based on the out-of-bag predictions of the random forest strategy. Lastly, the R-square value of the model fitting and a 10-fold cross-validation approach was applied to select the model and evaluate its predictive performance.

In clinical practice, patients were biopsied only once, and the receptivity status was identified as pre-receptive, receptive, and post-receptive. Subsequently, individual optimal implantation points for each patient were predicted with hourly precision using a random forest regression model.

### 2.4 Outcome measures

The primary outcome was the clinical pregnancy rate, while secondary outcomes included the rates of positive human chorionic gonadotropin (hCG) levels and biochemical pregnancies. Clinical pregnancy was defined as the confirmation of an intrauterine gestational sac with a foetal heartbeat on ultrasound, while positive hCG was defined as an hCG level >10 mIU/mL. Biochemical pregnancy was defined as a decrease in serum hCG levels following a positive pregnancy test in the absence of a gestational sac on ultrasonography.

### 2.5 Statistical analysis

Normally distributed continuous data are expressed as mean ± standard deviation and were compared using independent samples t-tests. Conversely, non-normally distributed continuous data are expressed as median and interquartile range and were compared using the Mann–Whitney U test. Finally, categorical data are expressed as counts and percentages and were compared using the chi-square test. To further verify the results, logistic regression models were conducted, and odds ratio (OR) and its 95% CI before and after adjusting for confounders were calculated. Statistical significance was set at P<0.05. Statistical analyses were performed using IBM SPSS software version 26.0 (Armonk, NY: IBM Corp.).

## 3 Results

### 3.1 Baseline clinical characteristics of the study participants

A total of 387 patients with RIF were recruited for this study. Group rsERT comprised 115 patients who underwent endometrial biopsy and rsERT-guided pET, while Group FET comprised 272 patients who underwent standard FET without rsERT. The baseline clinical characteristics of the recruited patients are displayed in **Table 1**. Group rsERT had more previously failed embryo transfer cycles than Group FET; however, other characteristics—including maternal age, body mass index, use of the GnRH-a HRT cycle, number of transferred embryos, endometrial thickness, and embryo stage—were comparable between the groups. Additionally, all patients in this study underwent transfer with at least one high-quality blastocyst.

**Table 1:** Baseline clinical characteristics of the participants in both groups.

Characteristic	Group ERT (n=115)	Group FET (n=272)	P-value
Maternal age, mean ± SD, y	32.7 ± 3.4	33.2 ± 4.2	0.291
No. of previous failed cycles, median (IQR)	2 (2-3)	2 (2-2)	**0.003**
BMI, mean ± SD, kg/m^2^	22.1 ± 3.9	21.9 ± 3.2	0.725
GnRH-a+HRT (n, %)	10 (8.7%)	34 (12.5%)	0.281
No. of transferred embryos, median (IQR)	2 (1-2)	1 (1-2)	0.086
Endometrial thickness, mean ± SD, mm	9.58 ± 1.18	9.79 ± 1.58	0.184
Embryo stage			0.582
D5 blastocysts (n, %)	96 (83.5%)	233 (85.7%)	
D6 blastocysts (n, %)	19 (16.5%)	58 (15%)	

ERT, endometrial receptivity test; FET, frozen embryo transfer; SD, standard deviation; IQR, interquartile range; BMI, body mass index; GnRH-a+HRT; gonadotropin-releasing hormone agonist hormone replacement therapy.

The bold values mean P<0.05.

### 3.2 rsERT results in patients with RIF

A total of 115 next-generation sequencing libraries were constructed for RNA-Seq using endometrial biopsy samples from the Group rsERT patients. The results showed that 39.1% (45/115) of patients who underwent rsERT were receptive, while 60.9% (70/115) were non-receptive, indicating WOI displacement. In the present study, all WOI displacements involved delay.

### 3.3 Clinical practice of rsERT-guided pET

**Figure 1:**
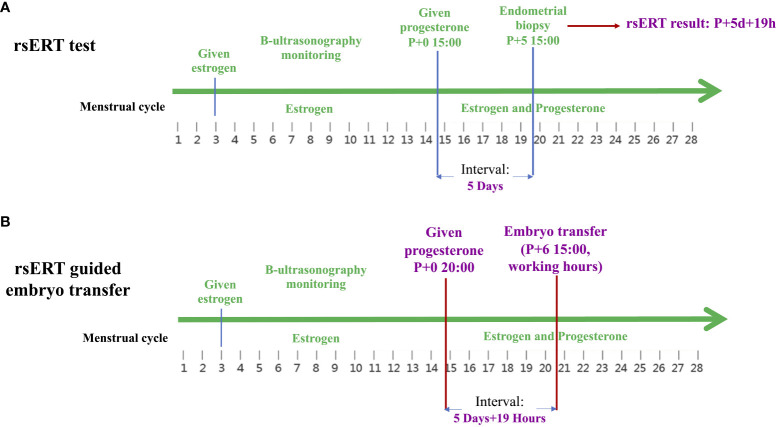
Clinical practice of rsERT-guided pET. **(A)** The rsERT results of the patient’s endometrial biopsy sample show that the improved optimal WOI period for blastocyst transfer was 5 days and 19 h after progesterone supplementation. **(B)** In the next blastocyst transfer cycle of the same patient, the peak administration for the first progesterone injection is calculated as 20:00 on the initial day instead of 15:00, and embryo transfer is performed at 15:00 on day 6 after progesterone supplementation. pET, personalized embryo transfer; rsERT, RNA-Seq-based endometrial receptivity test; WOI, window of implantation.

pET was performed at the time of the optimal WOI predicted by rsERT, which was accurate to the hour. As an example, the case of a blastocyst transfer cycle in a young patient is presented. This patient had previously undergone three FET cycles with morphologically high-quality blastocysts, among which the HRT cycle and natural ovulatory cycle were attempted, but clinical pregnancy was not achieved. After hysteroscopy revealed no endometrial abnormalities, the patient underwent rsERT to determine the optimal implantation point. As shown in **Figure 1**, the rsERT results of the patient’s endometrial biopsy sample indicated that the WOI was delayed and that the improved optimal WOI period for blastocyst transfer was 5 days and 19 h after progesterone supplementation. In response, for the next blastocyst transfer cycle, the peak administration for the first progesterone injection was calculated as 20:00 on the initial day instead of 15:00, and embryo transfer was performed at 15:00 on day 6 after progesterone supplementation. Consequently, precise rsERT results guided by pET were achieved.

### 3.4 rsERT-guided pET improves pregnancy outcomes in patients with RIF

Data on clinical outcomes were collected and analysed for patients in the rsERT and FET groups. The results showed that 73 of the 115 patients in Group rsERT showed positive hCG levels, of which 10 had biochemical pregnancies, and 63 achieved clinical pregnancies (**Table 2**). For Group FET patients, 140 of 272 had positive hCG levels, 35 of 140 had biochemical pregnancies, and the remaining 105 achieved clinical pregnancy (**Table 2**). These results demonstrated that Group rsERT patients, who underwent ERT-guided pET, achieved a significantly higher positive hCG (63.5% vs. 51.5%, P=0.03) and clinical pregnancy rates (54.8% vs. 38.6%, P=0.003) than Group FET patients (**Table 2**). And a lower biochemical pregnancy rate (13.7% vs. 25%, P=0.055) was found in Group rsERT patients, although this difference was not statistically significant (**Table 2**). All these findings were consistent with the results from the logistic regression analysis adjusted for all confounding factors including maternal age, BMI, No. of previous failed cycles, No. of transferred embryos, endometrial thickness.

**Table 2:** Clinical outcomes of patients who underwent rsERT vs. those who did not.

Variable	Group ERT, n (%) (n=115)	Group FET, n (%) (n=272)	OR (95%CI)	Adjusted OR (95%CI)^*^
**Positive hCG**	73 (63.5%)	140 (51.5%)	1.64^a^ (1.05-2.57)	1.73^a^ (1.02-2.96)
**Biochemical pregnancy**	10/73 (13.7%)	35/140 (25.0%)	0.476 (0.221-1.028)	0.54 (0.21-1.36)
**Clinical pregnancy**	63 (54.8%)	105 (38.6%)	1.93^a^ (1.24-3.00)	1.88^a^ (1.11-3.18)

rsERT, RNA-Seq-based endometrial receptivity test; ERT, endometrial receptivity test; FET, frozen embryo transfer; hCG, human chorionic gonadotropin.

^*^Adjusting for maternal age, BMI, No. of previous failed cycles, No. of transferred embryos, endometrial thickness.

a Statistically significant, with P < 0.05.

In Group rsERT, 39.1% (45/115) of patients were receptive, while 60.9% (70/115) were non-receptive. Among the receptive patients, 28 of 45 showed positive hCG results, 6 of 28 had biochemical pregnancies, and the remaining 22 had clinical pregnancies (**Table 3**). Compared with Group FET patients, receptive patients guided by rsERT with hourly precision showed a higher clinical pregnancy rate (48.9% vs. 38.6%, P=0.192) and a lower biochemical pregnancy rate (21.4% vs. 25%, P=0.688), although neither were statistically significant (**Table 3**).

**Table 3:** Clinical outcomes of receptive patients who underwent transfer with and without rsERT.

Variable	Receptive, n (%) (n=45)	Group FET, n (%) (n=272)	OR (95%CI)	Adjusted OR (95%CI)^*^
Positive hCG	28 (62.2%)	140 (51.5%)	1.55 (0.81-2.97)	1.55 (0.71-3.39)
Biochemical pregnancy	6/28 (21.4%)	35/140 (25.0%)	0.82 (0.31-2.18)	0.91 (0.26-3.22)
Clinical pregnancy	22 (48.9%)	105 (38.6%)	1.52 (0.81-2.87)	1.42 (0.65-3.09)

rsERT, RNA-Seq-based endometrial receptivity test; FET, frozen embryo transfer; hCG, human chorionic gonadotropin.

^*^Adjusting for maternal age, BMI, No. of previous failed cycles, No. of transferred embryos, endometrial thickness.

Among the non-receptive patients, 45 of 70 had positive hCG levels, 4 of 45 had biochemical pregnancy, and 41 achieved clinical pregnancy (**Table 4**). These results indicated that non-receptive patients had a significantly higher clinical pregnancy rate (58.6% vs. 38.6%, P=0.003) than Group FET patients (**Table 4**), and this difference was still statistically significant after adjusting for confounding factors. A lower biochemical pregnancy rate (8.9% vs. 25%, P=0.021) was found in Group rsERT patients, but this difference was not statistically significant after the adjustment (Adjusted OR 0.36, 95%CI: 0.10-1.28) (**Table 4**). Furthermore, receptive and non-receptive patients were compared; the latter had a higher probability of achieving clinical pregnancy (58.6% vs. 48.9%, P=0.309) and a lower risk of biochemical pregnancy (8.9% vs. 21.4%, P=0.13), although this difference was not statistically significant.

**Table 4:** Clinical outcomes of non-receptive patients who underwent transfer with and without rsERT.

Variable	Non-receptive, n (%) (n=70)	Group FET, n (%) (n=272)	OR (95%CI)	Adjusted OR (95%CI)^*^
Positive hCG	45 (64.3%)	140 (51.5%)	1.70 (0.99-2.92)	1.93^a^ (1.00-3.71)
Biochemical pregnancy	4/45 (8.9%)	35/140 (25.0%)	0.29^a^ (0.10-0.88)	0.36 (0.10-1.28)
Clinical pregnancy	41 (58.6%)	105 (38.6%)	2.25^a^ (1.32-3.84)	2.24^a^ (1.18-4.24)

rsERT, RNA-Seq-based endometrial receptivity test; FET, frozen embryo transfer; hCG, human chorionic gonadotropin.

^*^Adjusting for maternal age, BMI, No. of previous failed cycles, No. of transferred embryos, endometrial thickness.

a Statistically significant, with P < 0.05.

## 4 Discussion

This retrospective study aimed to provide important information on the efficacy of rsERT, which can predict the optimal implantation point with hourly precision. Patients who received embryo transfer during their individual WOI predicted by rsERT were compared with those who underwent conventional FET, which demonstrated that rsERT-guided pET significantly improved reproductive outcomes in patients with RIF.

RIF is an intractable problem that affects both physicians and patients during ART treatment. Recently, several therapies have been proposed as potential treatments for patients with RIF (8); these include those aiming to adjust the maternal immune system and endometrial environment, such as using low-molecular-weight heparin, immunosuppressors, and uterine instillation (24, 25), as well as PGT-A, which can promote the selection of euploid embryos (2). However, there is still a lack of effective and objective methods to resolve the asynchrony between embryo release and endometrial readiness, which has been detected in a significant proportion of patients with RIF (9, 26, 27).

The concept of pET was introduced to improve treatment outcomes of patients with RIF by synchronizing the timing of embryo transfer and the period of endometrial receptivity (12). Considering its ability to determine the receptivity of an endometrial sample and predict the individual WOI of each patient (12), ERA has been continuously studied in the general infertile population and in patients with previous failed embryo transfers via retrospective studies, in both prospective studies and randomized clinical trials (RCTs). All these results suggested that the routine clinical application of ERA was precluded in the general infertile population, due to ERA and pET not being more effective than standard ET (16, 17, 19, 28–32). However, uncertainties remained regarding whether ERA can improve clinical outcomes in RIF patients (12, 15, 19, 27, 30, 33–42). Therefore, we could conclude that ERA and pET present no or limited effectiveness in good prognosis patients, and whether ERA could benefit RIF patients still need more high-quality RCTs.

To improve the reproductive outcomes of patients with RIF, He et al. established a novel rsERT model using RNA-Seq to accurately predict the optimal WOI and identify biomarkers for endometrial receptivity (21). Based on the 175 markers selected from the DEGs, rsERT predicted the optimal implantation point with hourly precision through the application of the random forest algorithm. Compared with ERA—which enables clinicians to identify endometrial receptivity transition phases with 12-hour shifts—rsERT can predict the optimal implantation point with hourly precision. Although with improvements in methodologies, more studies on the clinical utility of rsERT are needed. In a prior study, rsERT significantly improved the implantation rate in patients with RIF who had D3 embryos transferred (21). Although the sample size was limited and such differences did not appear in patients with blastocysts transferred, a novel technology that can predict WOI with such high accuracy is expected to improve pET effectiveness.

Considering the difficulties in determining the specific time of the LH surge and ovulation in the natural cycle, the HRT/GnRH-a HRT cycle for endometrial preparation was performed to match the high accuracy of rsERT; furthermore, all patients received intramuscular progesterone as supplementation to ensure accurate timing of the first progesterone administration. Thus, a WOI displacement rate of 60.9% was detected, which is much higher than the previous results detected using rsERT (21, 43), and all displacements were delayed. In terms of clinical outcomes, rsERT was demonstrated to significantly improve the probability of patients with RIF achieving clinical pregnancy in the subsequent FET cycle, particularly in those with a displaced WOI. Although not statistically significant, patients with normal receptive results whose embryo transfer was guided by the WOI with the hourly precision predicted from rsERT still showed a clinical pregnancy rate that was 10% higher (48.9% vs. 38.6%) than that in patients who underwent standard FET. This difference indicates that a non-displaced but narrow WOI may be a cause of RIF, which cannot be detected by ERA in some patients; however, the ability of rsERT to predict the optimal WOI with hourly precision may improve clinical outcomes in these patients.

The strengths of the study include a relatively large sample size, the inclusion of patients who underwent HRT or GnRH-a HRT cycles for endometrial preparation, and the fact that all subjects underwent hysteroscopy before embryo transfer to ensure the absence of uterine cavity lesions. Additionally, the endometrial receptivity prediction model of rsERT was optimized for one-point rather than three-point sampling prediction at the beginning of the design (21), which significantly improved the clinical feasibility of this technique. The one-point sampling model included endometrial samples with clinical pregnancy guided by a previous three-point rsERT method in both HRT and natural cycles, making the test available for application in HRT cycles. However, one caveat was that the sample size of the machine-learning model was limited. Model upgrades and iterations are needed for further study. The other limitation of this study was the retrospective study design and morphological criteria used to select embryos for transfer, which may have introduced bias due to uncertainty regarding embryonic euploidy. Furthermore, our study only included patients with RIF; as such, it is uncertain whether rsERT can improve clinical outcomes in unselected infertile patients. Further studies are required to evaluate the efficacy of rsERT in unselected infertile patients with euploid embryo transfer.

In conclusion, rsERT-guided pET significantly improved the reproductive outcomes of patients with RIF, preliminarily validating the clinical effectiveness of this novel procedure for endometrial evaluation.

## Data availability statement

The datasets presented in this study can be found in online repositories. The names of the repository/repositories and accession number(s) can be found below: https://www.ncbi.nlm.nih.gov/geo/, GSE24547.

## Ethics statement

The studies involving humans were approved by the institutional review board of the Boai Hospital of Zhongshan (Application ID: KY-2022-010-14; date of approval: October 2022). The studies were conducted in accordance with the local legislation and institutional requirements. The participants provided their written informed consent to participate in this study.

## Author contributions

YX: Data curation, Formal analysis, Methodology, Writing – original draft, Writing – review & editing. JD: Data curation, Formal analysis, Investigation, Methodology, Software, Writing – original draft. YZ: Conceptualization, Data curation, Methodology, Software, Writing – review & editing. XLL: Methodology, Project administration, Writing – review & editing. YC: Writing – original draft, Writing – review & editing. LM: Data curation, Project administration, Writing – review & editing. SJ: Data curation, Project administration, Writing – review & editing. XFL: Conceptualization, Funding acquisition, Supervision, Visualization, Writing – review & editing.
